# High expression of the HMGB1–TLR4 axis and its downstream signaling factors in patients with Parkinson's disease and the relationship of pathological staging

**DOI:** 10.1002/brb3.948

**Published:** 2018-03-25

**Authors:** Yi Yang, Chenyang Han, Li Guo, Qiaobin Guan

**Affiliations:** ^1^ Department of Neonatologe The Second Affiliated Hospital of Jiaxing University Jiaxing China

**Keywords:** high‐mobility group box protein 1, myeloid differentiation factor 88, nuclear factor kappa B, Parkinson's disease, toll‐like receptor 4, tumor necrosis factor alpha

## Abstract

**Objective:**

To detect the expression of high‐mobility group box protein 1 (HMGB1) and toll‐like receptor 4 (TLR4) and their downstream signaling factors—myeloid differentiation factor 88 (MyD88), nuclear factor kappa B (NF‐κB), and tumor necrosis factor alpha (TNF‐α)—in the sera of patients with Parkinson's disease (PD) in order to evaluate the relationship of the HMGB1–TLR4 axis with PD development and progression.

**Methods:**

The serum HMGB1 and TLR4 protein levels of 120 patients with PD and 100 healthy volunteers were measured using double‐antibody sandwich ELISA, and their correlations with PD staging, disease duration, drug treatment effectiveness, and clinical classification were analyzed. In addition, their correlations with the key downstream factors of the HMGB1–TLR4 axis (MyD88, NF‐κB, and TNF‐α) were analyzed.

**Results:**

HMGB1 and TLR4 expressions were higher in the peripheral blood of patients with PD than in healthy volunteers. PD patients with poor drug treatment outcomes had significantly higher HMGB1 and TLR4 expressions than PD patients with stable drug treatment outcomes. Higher HMGB1 and TLR4 expressions were found in patients at higher PD stages, and patients with >4‐year disease duration had significantly higher HMGB1 and TLR4 expressions than patients with <4‐year disease duration. No significant difference in HMGB1 and TLR4 expressions was found among patients with tremor‐dominant, akinetic‐rigid, and mixed subtypes of PD. NF‐κB and TNF‐α expressions were positively correlated with high expression of the HMGB1–TLR4 axis.

**Conclusion:**

High expression of the HMGB1–TLR4 axis is closely associated with PD development, progression, drug treatment effectiveness, staging, and disease duration and has great significance for PD diagnosis and treatment.

## 1 BACKGROUND

Parkinson's disease (PD) is a common neurological disorder in the elderly, with the basic pathological feature of the loss of substantia nigra and striatum dopaminergic neurons and the clinical manifestations of resting tremor, bradykinesia, muscle rigidity, and abnormal posture and pace (Zesiewicz, Sullivan, & Hauser, 2017). The pathogenesis of PD has not been entirely clarified. Previous research has shown that PD is significantly associated with genetic factors, environmental factors, aging factors, and the depletion of mitochondrial function (Pfeiffer, 2006). In recent years, neuritis has been found to play an important role in the development and progression of PD (Kones, 2010; Rees et al., 2011). As an extracellular high‐mobility group family protein, high‐mobility group box 1 (HMGB1) protein not only initiates and increases the inflammatory immune response but also plays an important role in brain tissues (Cheng et al., 2017).

HMGB1 in the nucleus is transferred to receptor for advanced glycation end products (RAGE) through passive release and active release to extracellular target cells (Herold et al., 2007). RAGE expressed on endothelial cells, monocytes, macrophages, and other cells surfaces. After combining with HMGB1, it mediated the activation of NF‐kB, JAK/STAT, and MAPK family. The main receptors of HMGB1 on the surface of macrophages have been found to be TLR2 and TLR4, and TLR4 played an important role in neural disease (Ohtsu et al., 2017). High HMGB1 expression promotes the activation of astrocyte RAGE–MAPK signaling, which in turn promotes the expression of chemokines, cyclooxygenase 2, matrix metalloproteinase 9, and many other bioactive molecules (Karuppagounder et al., 2014), especially those involved in neuroinflammation (Karuppagounder et al., 2014). A previous study showed that HMGB1 induced the expression of interleukin (IL)‐6 and other inflammatory cytokines in brain tissues (Kiiski et al., 2017). Expression of this neuroinflammatory cytokine promotes neuron apoptosis and increases the development and progression of neurodegenerative disease in the central nervous system. And HMGB1 also regulated the release of excitatory neurotransmitters. Pedraai suggested (Pedrazzi et al., 2006) that HMGB1 also promotes the release of endogenous glutamic acid and D‐aspartic acid in vitro from glial cells. In the study of AD, the expression of HMGB1 was coexisting with the senile plaques induced by β‐amyloid peptide (Aβ). It can promote the binding of Aβ and inhibit the phagocytosis and removal of microglia (Frank et al., 2015). In summary, the expression of HMGB1 is found in the brain. At present, HMGB1 is found to play an important role in many kinds of nervous system diseases, but rarely reported in PD. This study evaluated the relationship between the expression of HMGB1 and its receptor, toll‐like receptor 4 (TLR4), and the development and progression of PD.

## 2 MATERIALS AND METHODS

### 2.1 Case selection

A total of 120 patients who visited and were diagnosed with PD in our hospital (The Second Affiliated Hospital of Jiaxing University, Jiaxing 314000, China) between June 2015 and June 2017 were included in this study. The inclusion criteria for the patients with PD were as follows: (1) had bradykinesia—slow initiation of voluntary movement and progressive reduction in speed and amplitude of repetitive motion once the disease had progressed; (2) had one of the following symptoms: muscle stiffness, resting tremor (4–6 Hz), and postural instability (not due to primary vision, vestibular, cerebellum, or ontological sensory dysfunction).

The exclusion criteria were as follows: (1) had repeated strokes accompanied by stepwise progression of PD; (2) had a history of brain injury; (3) symptoms occurred due to psychotropic medication; (4) had unilateral symptoms 3 years after the disease onset; (5) had signs of a cerebellar lesion; (6) had Babinski's and other pathological signs (+); (7) had brain tumor(s) and other organic lesion(s) screened by computed tomography (CT) or magnetic resonance imaging (MRI); and (8) did not respond to high‐dose levodopa treatment.

Patients with PD included in the study also had at least three of the following auxiliary diagnoses: (1) unilateral onset; (2) resting tremor; (3) progressive disease; (4) asymmetries of the symptoms, more severe on the side of disease onset; (5) significant curative effect of levodopa (70–100% efficacy); (6) severe levodopa‐induced dysplasia; (7) levodopa effect lasted more than 5 years; and (8) more than 10 years of the disease course. All PD patients were ranked in accordance with the Hoehn and Yahr scale, confirming 40 patients at Stage I, 35 patients at Stage II, 35 patients at Stage III, 10 patients at Stage IV, and 0 patients at Stage V.

### 2.2 Control group

For this study, 100 healthy volunteers were selected from the physical examination center in our hospital during the same period as the normal controls, excluding those with infectious disease(s) and consumption of immunosuppressive agent(s) and hormone(s). All research subjects or their family members signed written informed consent forms before participating in the study (involving data and blood sample collection). The research protocol of this study was approved by the Ethics Committee.

### 2.3 Sample collection and detection

All patients with PD and healthy volunteers were fasted for 12 hr after hospital admission to collect 2 ml peripheral venous blood from each participant. The blood sample was then stored at room temperature for 30 min and centrifuged for 20 min to collect serum from the upper supernatant. All serum samples were preserved at −80°C for later experiments. A double‐antibody sandwich enzyme‐linked immunosorbent assay (ELISA) was used to detect the serum HMGB1–TLR4 axis, myeloid differentiation factor 88 (MyD88), nuclear factor kappa B (NF‐κB), and tumor necrosis factor alpha (TNF‐α) protein expression via the absorbance (A), measured at a 450 nm wavelength by a microplate reader. The HMGB1 Elisa Kit (Shino‐Test Corporation, Kanagawa, Japan, batch number: A18521), The TLR4, MyD88, NF‐κB, TNF‐α Elisa Kit (MSK Corporation, WUHAN, China, batch number: A1852115241, 17241, 25432, 17254).

The protein contents were calculated according to the standard curve using the curve fitting equation, with the standard concentrations on the *x*‐axis and corresponding A in each concentration of the standards on the *y*‐axis. The protein contents are presented in ng/L throughout the paper.

### 2.4 Statistical analysis

SPSS 21.0 software (SPSS Inc., Chicago, IL) was used to analyze the data using an independent sample *t* test and Pearson's correlation analysis. *p *<* *.05 was considered statistically significant.

## 3 RESULTS

### 3.1 Comparing general information between PD and control groups

The general information of the patients with PD (PD group, *n* = 120) and healthy volunteers (control group, *n* = 100), including age group, gender, underlying health problems, smoking history, and body weight, is shown in Table 1. There was no significant difference between the groups (*p *>* *.05).

**Table 1:** Comparison of the general data of the patients with Parkinson's disease (PD)

General data	PD group (%)	Control group (%)
Age (years)	67.5 ± 4.2	65.3 ± 5.1
≤60	32 (26.7)	29 (29)
>60	88 (73.3)	71 (71)
Gender		
Male	70 (58.3)	57 (57)
Female	50 (41.7)	43 (43)
Body weight	72.5 ± 7.3	71.3 ± 6.2
Smoking history	53 (44.2)	45 (45)
Hypertension case	73 (60.8)	58 (58)
Diabetes case	50 (41.7)	43 (43)
PD stages		
Stage I	40 (33.3)	
Stage II	35 (29.2)	
Stage III	35 (29.2)	
Stage IV	10 (8.3)	
Stage V	0 (0)	

### 3.2 Comparing serum HMGB1 and TLR4 levels between PD and control groups

**Figure 1:**
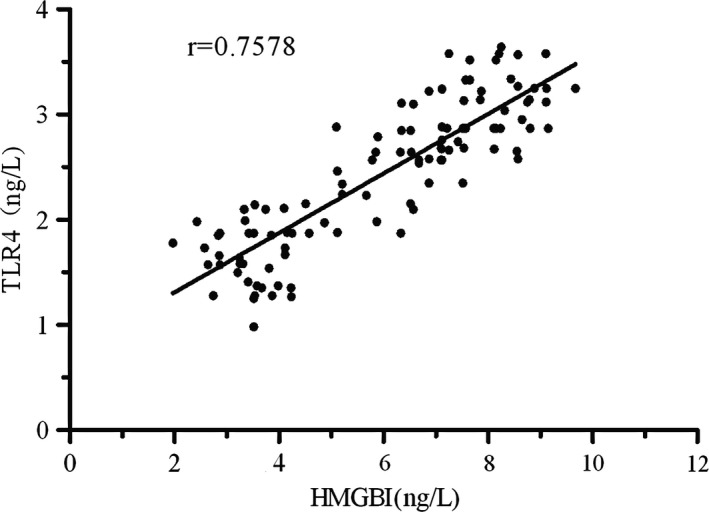
Correlation between HMGB1 and TLR4 expressions in the peripheral blood of patients with PD

The HMBG1 level in the peripheral blood was significantly higher in the PD group (5.96 ± 2.1) than in the control group (1.3 ± 0.18, *p *<* *.05); similarly, the TLR4 level in the peripheral blood was significantly higher in the PD group (2.56 ± 0.78) than in the control group (0.87 ± 0.12, *p *<* *.05; Table 2). There was a correlation between HMGB1 and TLR4 expressions in the peripheral blood of patients with PD (*r *=* *.7578; Figure 1).

**Table 2:** Comparison of serum HMGB1 and TLR4 levels in patients with Parkinson's disease (PD) and healthy volunteers (mean ± standard deviation, ng/L)

Group	Cases	HMGB1	TLR4
Control group	100	1.3 ± 0.18	0.87 ± 0.12
PD group	120	5.96 ± 2.1	2.56 ± 0.78
*t* value		22.7	21.2
*p* value		<.0001	<.0001

### 3.3 Comparing serum HMGB1 and TLR4 levels of PD patients at different stages of the disease

**Figure 2:**
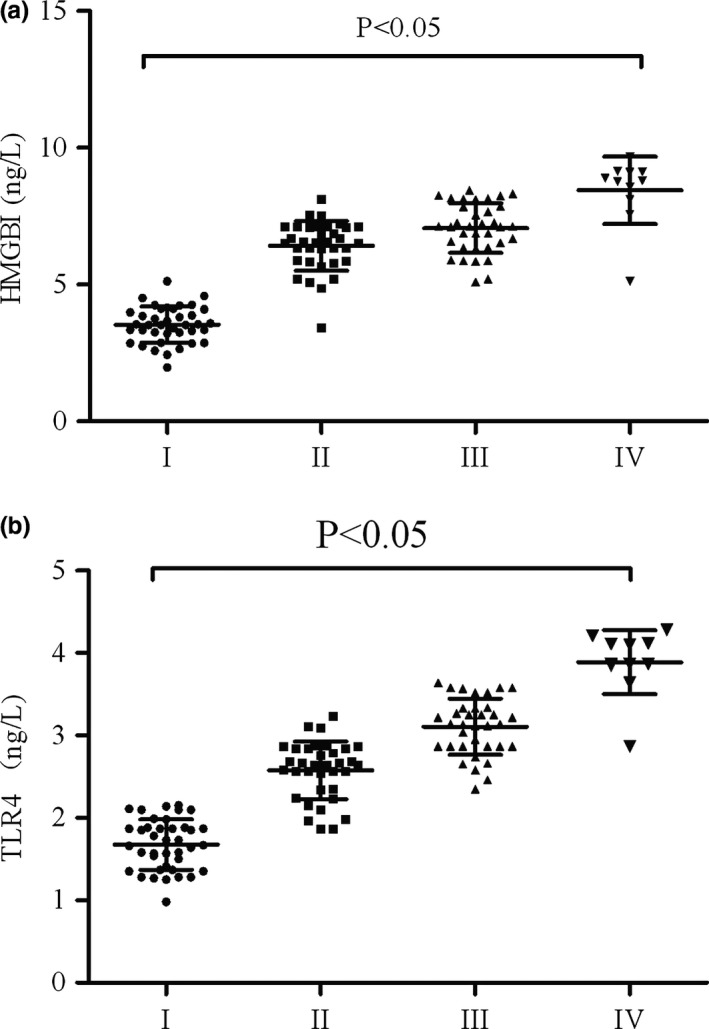
Relationship between serum HMGB1 and TLR4 expressions and PD staging of patients with PD. (a) Serum HMGB1 expression in patients with PD at different PD stages. Serum HMGB1 expression was increased with increasing PD stages. Significant differences in HMGB1 expression were found among different PD stages (*p *<* *.05). (b) Serum TLR4 expression in patients with PD at different PD stages. Serum TLR4 expression was increased with increasing PD stages. Significant differences in TLR4 expression were found among different PD stages (*p *<* *.05)

The serum HMGB1 levels of the patients with PD at Stages I, II, III, and IV were 3.53 ± 1.01, 6.41 ± 0.87, 7.16 ± 0.68, and 8.44 ± 1.12, respectively, which were significantly higher than those of the healthy volunteers (*p *<* *.05). The serum TLR4 levels of the patients at Stages I, II, III, and IV were 1.67 ± 0.40, 2.57 ± 0.51, 3.11 ± 0.42, and 3.89 ± 0.61, respectively, which were also higher than those of the healthy volunteers (*p *<* *.05). The serum HMGB1 and TLR4 protein expressions increased with increasing PD stages, suggesting that HMGB1 and TLR3 expressions reflect the disease progress and severity of PD to some extent (Figure 2).

### 3.4 Relationship between serum HMGB1 and TLR4 levels and the clinical classification of PD patients

We divided the clinical manifestations of the 120 patients with PD into mixed (*n* = 48), tremor‐dominant (*n* = 38), and akinetic‐rigid (*n* = 34) subtypes. PD patients with the mixed subtype mainly had the clinical manifestations of limb tremor and muscle rigidity, with serum HMGB1 and TLR4 levels of 5.12 ± 1.07 and 2.43 ± 0.87, respectively. PD patients with the tremor‐dominant subtype mainly had the clinical manifestation of limb tremor without obvious muscle rigidity and had serum HMGB1 and TLR4 levels of 5.08 ± 0.96 and 2.52 ± 0.72, respectively. Patients with PD in the akinetic‐rigid subtype mainly had the clinical manifestation of muscle rigidity, with serum HMGB1 and TLR4 levels of 5.18 ± 1.37 and 2.38 ± 0.80, respectively. No significant difference in HMGB1 and TLR4 expressions was found between groups (*p *>* *.05), suggesting that HMGB1 and TLR4 expressions have no correlation with the clinical manifestation of PD (Table 3).

**Table 3:** Correlation between serum HMGB1 and TLR4 expressions and clinical classification of patients with Parkinson's disease (mean ± standard deviation ng/L)

Group	Cases	HMGB1	TLR4
Mixed subtype	48	5.12 ± 1.07	2.43 ± 0.87
Tremor‐dominant subtype	38	5.08 ± 0.96	2.52 ± 0.72
Akinetic‐rigid subtype	34	5.18 ± 1.37	2.38 ± 0.80
*p*		>.05	>.05

Comparison between groups, *p* > .05.

### 3.5 Correlation between serum HMGB1 and TLR4 expressions and the efficacy of PD drug therapy in PD patients

All 120 patients with PD received combination therapy of dopaminergic agents and their inhibitors:(The dopaminergic agents used medopa which included levodopa 200 mg and benserazide 50 mg or sinemet which included carbidopa 25 mg and levodopa 100 mg. The inhibitors used selegiline or entacapone. The medopa made in Shanghai Roche Pharmaceuticals Ltd, the sinemet made in Hangzhou MSD Pharmaceuticals Ltd, the selegiline and entacapone made in Orion Corporation of Finland, and all of drugs were got from the hospital). Eighty‐three patients were well controlled by the drugs and 37 patients were not, showing disease progression. After analyzing the serum HMGB1 and TLR4 expressions, the PD patients with good therapeutic outcomes (effective group) were found to have serum HMGB1 and TLR4 expressions of 4.25 ± 0.65 and 2.31 ± 0.87, respectively, which were significantly lower than those of the PD patients with poor therapeutic outcomes (refractory group), who had serum HMGB1 and TLR4 expressions of 6.43 ± 1.14 and 3.10 ± 0.93, respectively (*p *<* *.05, Table 4).

**Table 4:** Serum HMGB1 and TLR4 levels and drug treatment effectiveness in patients with Parkinson's disease (mean ± standard deviation, ng/L)

Group	Cases	HMGB1	TLR4
Effective group	83	4.25 ± 0.65	2.31 ± 0.87
Refractory group	37	6.43 ± 1.14	3.10 ± 0.93
*t* value		8.3	6.7
*p* value		<.0001	<.0001

### 3.6 Relationship between serum HMGB1 and TLR4 expressions and the disease duration of PD patients

All 120 patients with PD were classified based on the duration of PD. There were 52 patients with <4 years of PD, 46 patients with 4–8 years of PD, and 22 patients with >8 years of PD. The serum HMGB1 and TLR4 expressions of the patients with <4‐year PD were 3.87 ± 0.85 and 1.85 ± 0.49, respectively. The serum HMGB1 and TLR4 expressions of the patients with 4–8 years of PD were 5.41 ± 0.76 and 2.52 ± 0.63, respectively. The serum HMGB1 and TLR4 expressions of the patients with >8‐year PD were 6.13 ± 1.12 and 3.13 ± 0.86, respectively. Significant differences in HMGB1 and TLR4 expressions were found between the groups (*p *<* *.05), suggesting that the prolonged duration of PD was positively associated with increased serum HMGB1 and TLR4 expressions (Table 5).

**Table 5:** Correlation between serum HMBG1 and TLR4 expressions and disease duration of patients with Parkinson's disease

Group	Cases	HMGB1	TLR4
<4‐year disease duration	52	3.87 ± 0.85	1.85 ± 0.49
4–8‐year disease duration	46	5.41 ± 0.76	2.52 ± 0.63
>8‐year disease duration	22	6.13 ± 1.12	3.13 ± 0.86
*p*		<.05	<.05

Comparison between groups, *p* < .05.

### 3.7 Expression of HMGB1–TLR4 axis downstream factors MyD88, NF‐κB, and TNF‐α in the sera of PD patients

**Figure 3:**
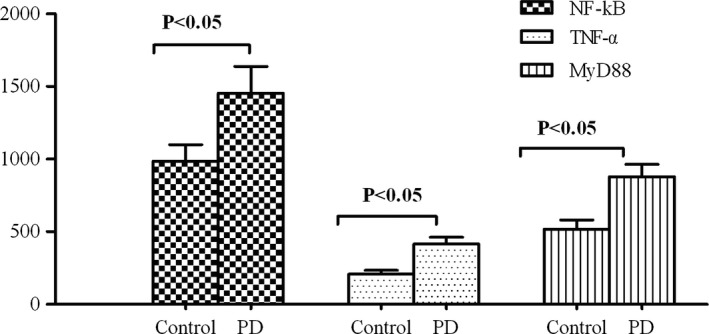
Expression of the HMGB1–TLR4 axis's downstream factors, MyD88, NF‐κB, and TNF‐α, in the sera of patients with PD

The serum MyD88, NF‐κB, and TNF‐α levels of the healthy controls were 518.2 ± 63.4, 985.3 ± 114.6, and 207.3 ± 27.4, respectively, which were significantly higher than those of the patients with PD (879.2 ± 85.3, 1453.4 ± 184.6, and 415.3 ± 46.7, respectively; *p *<* *.05), suggesting that HMGB1–TLR4 axis downstream signaling was positively associated with the high expression of the HMGB1–TLR4 axis (Figure 3).

## 4 DISCUSSION

HMGB1 is a non‐histone chromosome‐binding protein expressed in eukaryotic cells. It was first found to express in the brain and extensively express in heart, liver, spleen, lung, and kidney tissues (Gupta, Ghosh, & Nagarajan, 2013; Wang, Ward, & Sama, 2014). The extracellular HMGB1 is first found in the brain tissue, which is mainly related to immunoregulation. A variety of cells can release HMGB1, HMGB1 combined with the RAGE which expressed on the surface of macrophages and nerve cells to stimulate downstream signals. In‐depth research on HMGB1 has shown that HMGB1 is associated with TLR4‐mediated inflammatory response and a variety of diseases, such as sepsis and gliomas (Wang et al., 2015). The HMGB1–TLR4 axis is key to the inflammatory response; damaged cells and activated macrophages actively or passively release HMGB1, which induces the secretion of TNF‐α, IL‐6, and other inflammatory cytokines through signaling pathways. Early proinflammatory factors and HMGB1 itself promote the release of HMGB1 to form a loop, which amplifies the inflammatory response (Nair, Ebenezer, Saini, & Francis, 2015). TLR4 is the recognition receptor on the cell surface that identifies endogenous HMGB1. Activation of TLR4 recruits the adaptor protein MyD88, which can interact with IL‐1 and other receptor‐related kinase family proteins to form a complex that can eventually enable NF‐κB to enter nuclei and play regulatory roles (Wang et al., 2015; Zhang et al., 2005).

There are many external factors that induce PD and damage astrocytes and microglia in the human brain to stimulate the release of HMGB1 (Gao et al., 2011). The activated astrocytes or neurons stimulate HMBG1–TLR4 signaling through autocrine or paracrine signaling. An HMGB1‐based neuroinflammation study found HMGB1 to have a neurotrophic factor‐like effect (Li et al., 2016). Intracerebroventricular injection of HMGB1 was found to promote TNF‐α and IL‐6 expressions in mouse brain tissues (Fujita et al., 2016). The injection of HMGB1 into the mouse parietal lobe resulted in the partial elevation of iNOS and IL‐1β mRNA expressions 24 hr later. In addition, a study of Alzheimer's disease showed that HMGB1 stably binds to amyloid‐beta (Aβ) peptides to suppress Aβ phagocytosis by microglia (Mallama, Mccoysimandle, & Cianciotto, 2017). Therefore, HMGB1 plays a major role in promoting neuritis. NF‐κB, one of the downstream factors of HMGB1–TLR4 signaling, induces the expression of IL‐6 in macrophages. IL‐6, a pleiotropic proinflammatory factor, plays an important role in chronic inflammation and neuritis; TNF‐α is also involved in the inflammatory response by inducing NF‐κB activation. MyD88 mediates the activation of IL‐6 by activating a variety of toll‐like receptors (Liu et al., 2017). Therefore, the expressions of MyD88, NF‐κB, and TNF‐α reflect the severity of neuritis and the expression of the HMGB1–TLR4 axis.

This study examined the expression of the HMGB1–TLR4 axis and its downstream MyD88, NF‐κB, and TNF‐α factors in 120 patients with PD and 100 healthy volunteers to evaluate its relationship with PD staging, PD duration, and PD drug therapeutic outcomes. The expression of the HMGB1–TLR4 axis was significantly higher in the patients with PD than in the healthy volunteers, suggesting that the HMGB1–TLR4 axis plays an important role in PD pathogenesis. HMGB1 and TLR4 expressions increased with increasing PD stages. Higher PD staging indicated faster PD progress and higher severity, suggesting that sustained high‐level expression of the HMGB1–TLR4 axis was directly associated with the progression of PD. In addition, PD patients with poor PD‐drug therapeutic outcomes had significantly higher expression of the HMGB1–TLR4 axis than PD patients with good PD‐drug therapeutic outcomes. A previous study showed that injection of HMGB1 into the mouse brain increased p‐glycoprotein (p‐gp) expression in brain tissues (Chen et al., 2015). As a drug‐resistant protein, p‐gp may be one of the causes of poor PD‐drug therapeutic outcomes. Moreover, *in vitro* experiments have shown that HMGB1 can be used as one of the inflammatory cytokines to promote neuronal damage. Sathe, Mustafa, Martin, Tracey, & Teismann (2010), which directly confirms the neurotoxicity of HMGB1. In this study, we also found that the HMGB1–TLR axis downstream signaling factors, MyD88, NF‐κB, and TNF‐α, were highly expressed in the sera of patients with PD, indicating a connection between the upstream and downstream signaling of the HMGB1–TLR4 axis that plays an important role in the neuritis of PD.

## 5 CONCLUSION

In this study, we found high expression of the HMGB1–TLR4 axis and its downstream signaling factors in patients with PD, which was positively associated with PD staging and duration and correlated with PD‐drug therapeutic outcomes. In addition, the expression of the HMGB1–TLR4 axis upstream signaling factors was increased. High HMGB1–TLR4 axis expression promoted the development of neuritis, which may be one of the important factors in PD progression. This study provided a new reference target for the treatment of PD in the future. Our findings also showed that patients with longer PD duration have higher expression of the HMGB1–TLR4 axis.
